# Numerous chondritic impactors and oxidized magma ocean set Earth’s volatile depletion

**DOI:** 10.1038/s41598-021-99240-w

**Published:** 2021-10-22

**Authors:** Haruka Sakuraba, Hiroyuki Kurokawa, Hidenori Genda, Kenji Ohta

**Affiliations:** 1 Department of Earth and Planetary Sciences, Tokyo Institute of Technology, Ookayama, Meguro-ku, Tokyo, 152-8551 Japan; 2 Earth-Life Science Institute, Tokyo Institute of Technology, Ookayama, Meguro-ku, Tokyo, 152-8550 Japan

**Keywords:** Planetary science, Core processes, Early solar system, Geochemistry, Geodynamics, Inner planets

## Abstract

Earth’s surface environment is largely influenced by its budget of major volatile elements: carbon (C), nitrogen (N), and hydrogen (H). Although the volatiles on Earth are thought to have been delivered by chondritic materials, the elemental composition of the bulk silicate Earth (BSE) shows depletion in the order of N, C, and H. Previous studies have concluded that non-chondritic materials are needed for this depletion pattern. Here, we model the evolution of the volatile abundances in the atmosphere, oceans, crust, mantle, and core through the accretion history by considering elemental partitioning and impact erosion. We show that the BSE depletion pattern can be reproduced from continuous accretion of chondritic bodies by the partitioning of C into the core and H storage in the magma ocean in the main accretion stage and atmospheric erosion of N in the late accretion stage. This scenario requires a relatively oxidized magma ocean ($$\log _{10} f_{{\mathrm{O}}_2}$$ $$\gtrsim$$ $${\mathrm{IW}}$$$$-2$$, where $$f_{{\mathrm{O}}_2}$$ is the oxygen fugacity, $$\mathrm{IW}$$ is $$\log _{10} f_{{\mathrm{O}}_2}^{\mathrm{IW}}$$, and $$f_{{\mathrm{O}}_2}^{\mathrm{IW}}$$ is $$f_{{\mathrm{O}}_2}$$ at the iron-wüstite buffer), the dominance of small impactors in the late accretion, and the storage of H and C in oceanic water and carbonate rocks in the late accretion stage, all of which are naturally expected from the formation of an Earth-sized planet in the habitable zone.

## Introduction

Earth’s major volatile elements—carbon (C), nitrogen (N), and hydrogen (H)—are the main components of the atmosphere and oceans and the key elements for life. The budget of these major volatiles in the bulk silicate Earth (including the atmosphere, oceans, crust, and mantle: hereafter BSE) influences the volume of the oceans and the atmospheric inventory of C (${\mathrm{CO}}_2$) and N ($\hbox {N}_2$), and consequently, Earth’s habitable environment^1–3^. Similar isotopic compositions of volatiles in Earth and chondrites suggests that delivery was made chiefly by chondritic materials^4^. In contrast, although their absolute abundances are largely uncertain, the abundances of C–N–H in BSE relative to chondrites are known to have a V-shaped depletion pattern^5,6^. Owing to this discrepancy, the origin of the major volatile elements on Earth remains unclear^5^.

**Figure 1:**
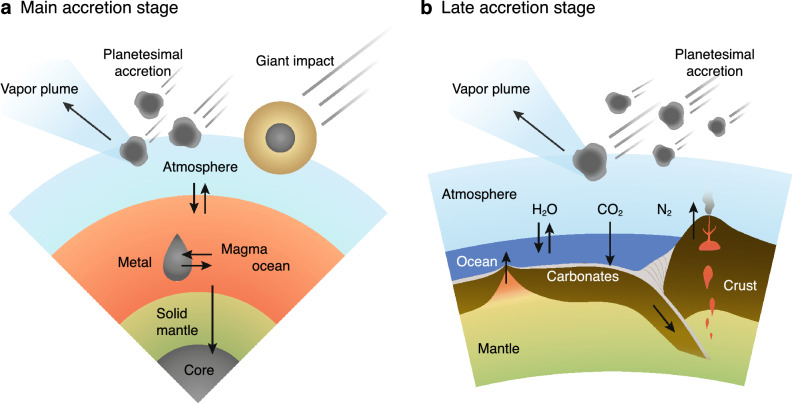
Cartoon of element partitioning processes during Earth’s accretion according to our model. Accreting planetesimals and giant impactors deliver volatiles and simultaneously form a vapour plume eroding the atmosphere. (**a**) Model for the main accretion stage (10% to 99.5% of the Earth’s mass). Equilibration among the magma ocean (silicate melt), liquid metal droplets transiting to the core, and the overlying atmosphere are achieved according to each metal silicate partitioning coefficient and solubility. (**b**) Model for the late accretion stage after the solidification of the magma ocean (the last 0.5%). We consider the liquid water oceans and the carbonate-silicate cycle to be driven by plate tectonics on the surface. In this stage, most H and C on Earth are stored in the oceans and carbonate rocks, respectively. Numerous impactors can selectively erode N.

The composition of major volatiles in the BSE should have been modified by element partitioning processes, including removal by core-forming metals and by atmospheric escape (Fig. 1). Although the origins and timing of volatile delivery are still debated, the delivery during the main accretion stage is expected based on the planet formation theory, which involves large-scale dynamic evolution, such as the Grand Tack model^7^. Recent isotopic analyses point to the late-accreting bodies being composed of enstatite chondrites^8^ or carbonaceous chondrites^9^, suggesting that volatile delivery continued into the late stage. On the growing proto-Earth with planetesimal accretion and several giant impacts, the formation of magma oceans allowed volatiles to be stored within the magma ocean^10^. Core-forming metal could have removed some of the iron-loving elements (siderophiles) from the magma ocean during the main accretion stage^11^. Volatiles partitioned into the atmosphere (atmophiles) were continuously removed via atmospheric erosion caused both by small planetesimal accretion^12^ and giant impacts^13^. The successive late accretion after the solidification of the magma ocean further removed and replenished volatile elements^14^.

Alhough several previous studies have attempted to explain the depletion patterns of major volatile elements in BSE^5,15,16^, the evolution of the volatile composition through the full accretion history has not been simulated. The previous studies employed ad hoc models where a single-stage metal-silicate equilibration event and complete/negligible atmospheric loss were assumed. Hirschmann^5^ showed that the combination of core segregation and atmospheric blow off would leave BSE with low C/H and C/N ratios compared with accreted material, and he concluded that the BSE’s high C/N ratio requires late accreting bodies with elevated C/N ratios compared with chondrites. Other works have attributed the discrepancy largely to the accretion of thermally processed or differentiated, non-chondritic bodies^15,16^, which are hypothetical and may not satisfy the isotopic constraints^8^. Here, we consider another mechanism that can fractionate C/N: the preferential loss of N relative to C and H by impacts during the late accretion stage, where N is partitioned into the atmosphere, while C and H are partitioned into the oceans and carbonate rocks^14^. This work builds on previous studies^5,15,16^ in terms of volatile element partitioning, but makes improvements to simulate core formation and atmospheric loss as continuous processes rather than single stage events.

**Figure 2:**
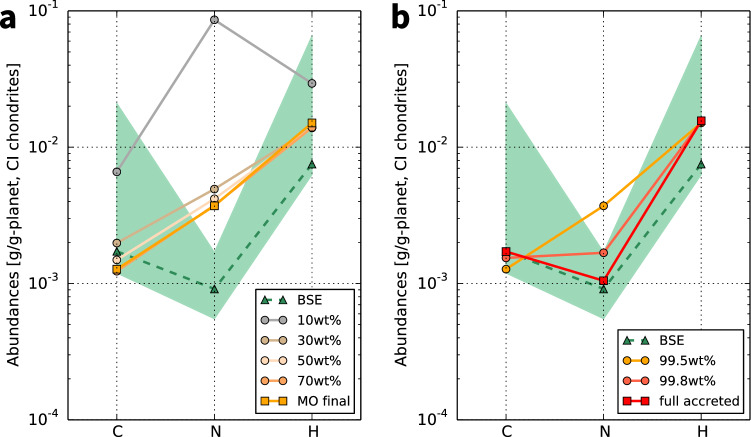
Evolution of major volatile abundances in the bulk silicate Earth (BSE) scaled by those of CI chondrites in the nominal model. The abundances are normalized by each planetary mass at each time for (**a**) the main accretion stage, from 10 to 99.5% of Earth’s accretion, and (**b**) the late accretion stage defined as the last 0.5% of accretion after the magma ocean solidification. The time sequence is shown by lines from top to bottom with snapshots. The thick orange and red lines correspond to the end of main and late accretion stages, respectively. The range in the current BSE composition estimate^4,5,17^ is shown for comparison (green area). The mean value of Hirschmann^5^ is shown as a reference for the relative depletion pattern (green line). See Table 1 for the composition of BSE and chondrites.

**Figure 3:**
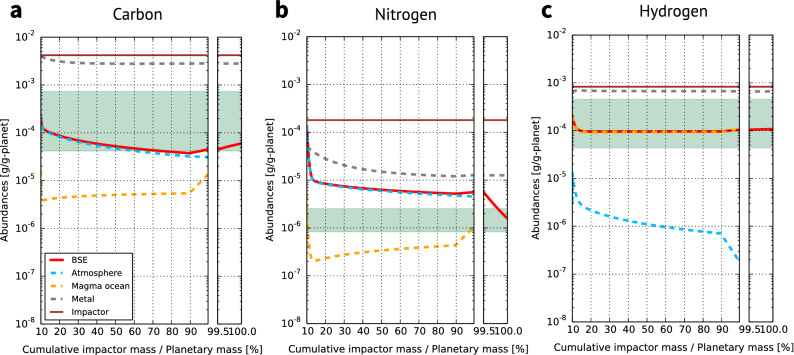
Evolution of the abundances of C, N, and H in the surface and interior reservoirs over the full accretion obtained from the nominal model. Dashed lines correspond to the amounts in the atmosphere (light-blue), magma ocean (orange), and metallic core (grey) for the main accretion phase and in the surface reservoirs (the atmosphere, oceans, and carbonate rocks: red solid line) for the late accretion phase. Solid lines mean the net cumulated into the bulk silicate Earth (BSE; red) and delivered by impactors (brown). The green areas denote the amounts in the current BSE. Plotted abundances are scaled by the planetary mass at a given time.

**Figure 4:**
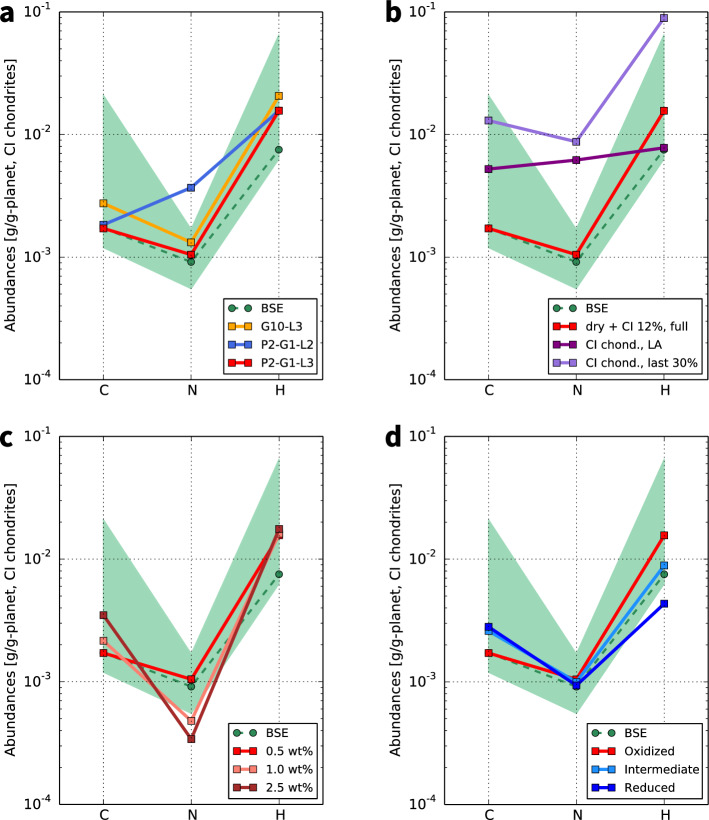
Dependence of final volatile composition of the bulk silicate Earth (BSE) on the accreting conditions. (**a**) Effects of the impactor’s size distribution. P2-G1-L3 (the nominal model, red line): planetesimal accretion ($q = 2$ and $q = 3$ for in the main and late accretion stages, respectively) and one giant impact. G10-L3 (orange line): ten giant impacts and the late accretion of planetesimals . P2-G1-L2 (blue line): shallower planetesimal size distribution ($q = 2$ throughout the full accretion) and one giant impact. Here we assumed $\mathrm {d}N/\mathrm {d}D\propto D^{-q}$, where *N*(*D*) is the number of objects of diameter smaller than *D*, and *q* is the power law index. (**b**) Dependence on volatile accretion scenarios. Late volatile accretion (dark-purple): volatiles are delivered only by late accretion with CI chondrite-like bodies. Heterogeneous accretion model (purple): volatiles are supplied in the last 30 wt% accretion with CI chondrite-like bodies. (**c**) Dependence on the late accretion mass. The mass of late accretion was varied from 0.5 wt% (brown) to 2.5 wt% (orange). (**d**) Dependence on the redox state of the magma ocean. Oxidized (the nominal model, red line), intermediate (light blue line), and reduced (blue line) conditions are compared. The solubilities and partitioning coefficients are summarised in Table 1.

In this study, we aimed to reproduce the V-shaped C–N–H pattern by considering realistic processes to the extent of today’s observational uncertainties. We modelled the evolution of the volatile abundances in the atmosphere, oceans, crust, mantle, and core through the full accretion by taking elemental partitioning and impact erosion into account. Figure 1 shows a schematic image of our model setting. The main and late accretion stages were modelled separately, and the masses of C, N, and H in each reservoir were computed using a multiple-boxes model (“Methods”). We assumed the existence of the oceans and the active carbonate-silicate cycle in the late accretion stage; the validity of this assumption is discussed. We explored the plausible accretion scenarios that reproduce the current BSE’s C–N–H composition pattern from the accretion of chondritic bodies. The major parameters were the size distribution of planetesimals in each stage; the number of giant impacts; the redox state of the magma ocean, which controls the solubility and metal-silicate partitioning coefficient of volatiles; the composition of impactors; and the total mass of the late accretion. Our nominal model, which is a successful case, assumes a volatile supply from CI chondrite-like building blocks, an oxidized magma ocean ($\log _{10} f_{{\mathrm{O}}_2}$ $\sim$IW+1, where $f_{{\mathrm{O}}_2}$ is the oxygen fugacity, $\mathrm{IW}$ is defined hereafter as $\log _{10} f_{{\mathrm{O}}_2}^{\mathrm{IW}}$, and $f_{{\mathrm{O}}_2}^{\mathrm{IW}}$ is $f_{{\mathrm{O}}_2}$ at the iron-wüstite buffer), a single giant impact, and a change in planetesimal size distribution with time, and 0.5 wt% late accretion. Figures 2 and 3 show the evolution of major volatile abundances for this successful case. As the composition of building blocks, a mixture of CI chondrite-like impactors (12 wt%) and dry objects (88 wt%) was fixed by exploring the best fit homogeneous accretion (see Supplementary Information). In order to understand the physical behaviours of the volatile element partitioning, we also calculated the evolution for other cases with different impactor size distributions, accretion scenarios, amounts of late accretion, and redox states of the magma ocean (Fig. 4). In the Supplementary Information, we show the results for the cases where we assume a different source for volatile elements (enstatite chondrites) and the range of partitioning coefficients and solubilities. We confirmed that other parameters such as the magma ocean depth, metal/silicate ratio, surface temperature during the magma ocean stage, and efficiency of impact erosion by a giant impact had only minor effects (Fig. S1). The uncertainties in the final volatile abundances in BSE caused by these minor parameters, except for the magma ocean depth, are smaller than 10%. As to the magma ocean depth, the uncertainties differ by species and the redox state of the magma ocean: a factor of $\sim$2 for H in the oxidized model, $\sim$40% for N and $\sim$15% for H in the reduced model, and smaller than 10% for the others (Fig. S1 for the oxidized model and the figure not shown for the reduced model).

## Origin of the V-shaped C–N–H depletion pattern

The V-shaped C–N–H depletion pattern compared with chondrites in the current BSE (Fig. 2) can be successfully reproduced from chondritic building blocks by considering both the element partitioning between reservoirs and the impact-induced atmospheric erosion simultaneously over all accretion stages. The successful case (the nominal model) assumed three conditions: an oxidized magma ocean (IW+1), a change in impactor size distribution with time, and the storage of H and C in oceanic water and carbonate rocks after magma ocean solidification. The nominal model assumed CI chondrite-like building blocks, but enstatite chondrite-like impactors’ case, where a smaller amount of H is present in the building blocks, also successfully reproduced the BSE composition under more limited conditions (see Supplementary Fig. S2, Fig. S3, Fig. S4, and Fig. S5). Element partitioning among the overlying atmosphere, magma ocean, and suspended metal droplets during the main accretion phase led to a subtle C deficit, N excess, and an adequate amount of H at the end of the main accretion stage (Fig. 2a). In the late accretion stage after solidification, the interplay of H and C storage in oceanic water and carbonate rocks and the preferential loss of N due to atmospheric erosion finally solved the remaining issue: N excess (Fig. 2b).

Earth’s C and H abundances were set chiefly during the main accretion stage (Fig. 3). Although the first kink of volatile abundances in each reservoir is set by the initial conditions (see “Methods”), the system soon evolves towards a quasi-steady state between the gain and loss of volatile elements. The highly siderophile property of C^18^ and high solubility of H^19^ in silicate melt under the oxidized condition in the nominal model caused those elements to be removed by core segregation. As the remaining part of C was partitioned into the atmosphere owing to its low solubility^20^, the atmospheric erosion led to C being more depleted than H in BSE. The low solubility of N^21^ in magma led to almost all N in BSE being partitioned into the atmosphere soon after the magma ocean solidification. For N, the impact-induced erosion governs the abundance evolution^14^, while the transport to the core governs for C and H. N also has a siderophile property, which has been proposed to cause N depletion in the BSE^16,22,23^. However, we found that partitioning into the core is less important compared with the atmospheric escape, even considering the uncertainty in the partitioning coefficient (see Supplementary Information). A Mars-sized, moon-forming giant impact^24^ was assumed in the nominal model, which corresponds to the kink at 90% Earth mass, but it did not modify the BSE volatile abundances significantly. We considered a completely molten mantle^25^ in the element partitioning after the giant impact, while a smaller molten fraction of 30 wt% was assumed for the planetesimal accretion. Thus, the larger mass of the magma ocean after the giant impact allowed increases in the abundances of all volatile elements in the magma ocean.

The abundance of N was decreased by approximately one order of magnitude during the late accretion phase owing to impact-induced atmospheric escape. The formation of oceans and the initiation of the carbonate-silicate cycle right after the solidification of the magma ocean trapped H and C into the surface reservoirs, and subsequently facilitated preferential N erosion from the atmosphere. Since N neither condense nor become incorporated into any solid or liquid reservoirs in our model, the final N abundance is determined by the balance between the supply by impactors and the loss by atmospheric erosion. From this result, we argue that the presence of oceans and carbonate formation in the late accretion stage are requirements to explain the current high C/N and H/N ratios of the BSE.

The exact timing of the ocean formation and the initiation of the carbonate-silicate cycle are unknown, but these assumptions are supported by theoretical, geological, and geochemical studies. As the timescale for the magma ocean solidification is short (0.2–7 Myrs after the last giant impact^26–28^), the oceans would have persisted in the late accretion stage. Archean sediments imply that the oceans^29^ and plate tectonics^30^ already existed at least 3.8 Gyr ago. Geochemical studies of Hadean zircons suggest the existence of oceans and the active plate boundary interactions in the Hadean^31,32^. Although plate tectonics might not have started on the Hadean Earth^33^, the storage of C by carbonate precipitation is possible even without plate tectonics as long as liquid water is present^34^. The continental crust, which releases water-soluble cations such as $\hbox {Ca}^{2+}$ and $\hbox {Mg}^{2+}$ on modern Earth, might not be present in the Hadean, but efficient carbonate formation is possible owing to seafloor weathering^35^. However, the presence of liquid water which allows silicate weathering to occur is required to drive the carbonate-silicate cycle^3^. Furthermore, if marine pH was neutral to alkaline ($>\sim$7), most of the total atmosphere plus ocean C inventory ($>\sim$90%) would have dissolved into the oceans as bicarbonate and carbonate ions^36^, as proposed for the preferential N loss by a giant impact^37^. We note, however, that a giant impact will vaporize the oceans, which could end up losing some C.

Another key assumption of our model is the slow (negligible) N fixation compared to C on early Earth during late accretion, which led to the preferential erosion of atmospheric N. A combined model of atmospheric and oceanic chemistry determined the lifetime of molecular N in anoxic atmospheres to be $>10^{9}$ years^38^. After Earth’s accretion ceased, N cycling between the atmosphere and mantle over a billion-year timescale^39^ would lead to lower N partial pressures in the later period (e.g., those recorded in the Archean^40^). In contrast, the timescale of carbonate precipitation even in the cation supply-limited regime ($\sim 10^6$ years^3^) is shorter than the duration of late accretion ($\sim 10^7$–$10^8$ years^41^).

## Size distribution of impactors and accretion models

**Table 1:** List of key parameters for the model calculations.

	C	N	H	C/N	C/H
BSE [μg/g-Earth]	42–730$^{\mathrm{a,b,c}}$	0.83–2.5$^{\mathrm{a,b,c}}$	44–450$^{\mathrm{a,b,c}}$	40 ± 8$^{\mathrm{a,d}}$	1.3 ± 0.3 $^{\mathrm{a,e}}$
CI chondrites [ppm]	20,000–50,000$^{\mathrm{f}}$	500–2,000$^{\mathrm{f}}$	4,700–10,500$^{\mathrm{g}}$	14.5 ± 2.5$^{\mathrm{d}}$	2–8$^{\mathrm{h}}$
CI model [ppm]	35,000	1,500	6,900	23	5
Enstatite chondrites [ppm]	2,000–7,000$^{\mathrm{f}}$	100–500$^{\mathrm{f}}$	90–600$^{\mathrm{i}}$	13.7± 12.1$^{\mathrm{d}}$	
EC model [ppm]	4,000	250	400	16	10
**Oxidised model**					
$S_{\mathrm{i}}$	1.6$^{\mathrm{j,k}}$	1.0$^{\mathrm{l}}$	M98 model$^{\mathrm{m}}$		
$D_{\mathrm{i}}^{\mathrm{met/sil}}$	500$^{\mathrm{n,o}}$	20$^{\mathrm{p}}$	6.5$^{\mathrm{q}}$		
**Intermediate model**					
$S_{\mathrm{i}}$	0.55$^{\mathrm{o}}$	5.0$^{\mathrm{p}}$	M98$\hbox {model}^{\mathrm{l}}$		
$D_{\mathrm{i}}^{\mathrm{met/sil}}$	1000$^{\mathrm{n,o}}$	20$^{\mathrm{p}}$	6.5$^{\mathrm{q}}$		
**Reduced model**					
$S_{\mathrm{i}}$	0.22$^{\mathrm{r}}$	50$^{\mathrm{l}}$	5.0$^{\mathrm{s}}$		
$D_{\mathrm{i}}^{\mathrm{met/sil}}$	3,000$^{\mathrm{n,o}}$	20$^{\mathrm{p}}$	6.5$^{\mathrm{q}}$		

Observational data and model reference values for the abundances of C, N, and H in the bulk silicate Earth (BSE) and chondrites (assumed as building blocks, CI chondrites and enstatite chondrites). We set CI chondritic model, solubilities $S_{\mathrm{i}}$ (the units are $({P_{\mathrm{i}}}/\mathrm{1 MPa})^{1/2} \,\mathrm{ppm}$ for reduced $S_{\mathrm{H}}$ and $\left( {P_{\mathrm{i}}}/\mathrm{1 MPa}\right) \,\mathrm{ppm}$ for the others, where $P_{\mathrm{i}}$ is the partial pressure of each molecule $\mathrm{i}$), and the partitioning coefficients $D_{\mathrm{i}}$ under each redox state by following Hirschmann^5^. (a) Hirschmann^5^, (b) Marty^4^, (c) Hirschmann^17^, (d) Bergin et al.^15^, (e) Hirschmann and Dasgupta^124^, (f) Grady and Wright^81^, (g) Vacher et al.^82^, (h) Kerridge^79^, (i) Piani et al.^84^, (j) Stolper and Holloway^123^, (k) Pan et al.^20^, (l) Libourel et al.^114^, (m) Moore et al.^19^, (n) Chi et al.^100^, (o) Armstrong et al.^101^, (p) Roskosz et al.^103^, (q) Okuchi^102^, (r) Wetzel et al.^112^, (s) Hirschmann et al.^67^.

Our results suggest the dominance of small (km-sized) impactors during the late accretion. The nominal model assumed a change in the size distribution of impactors from shallower ($q = 2$ in $\mathrm {d}N/\mathrm {d}D\propto D^{-q}$, where *N*(*D*) is the number of objects of diameter smaller than *D*, and *q* is the power law index) for the main accretion phase to steeper ($q = 3$, main asteroid belt-like) for the late accretion phase (see “Methods”, Fig. 4a). The impact erosion by the late accretion which has a shallow size distribution is not sufficient to reproduce the BSE’s N-depletion because km-sized small bodies are the most efficient in ejecting the atmosphere per unit mass of the impactor^12,42^.

The case where Earth formed by multiple giant impacts followed by late accretion also reproduced the current BSE volatile composition (Fig. 4a); however, atmospheric erosion by small bodies during late accretion is needed to obtain the V-shaped C–N–H pattern anyway. Since atmospheric loss per unit impactor mass is less efficient in giant impacts than in planetesimal accretion, larger amounts of volatiles remained in the BSE. In addition, incomplete mixing between the impactor’s core and magma ocean lowered the storage in the core.

The combination of a moon-forming giant impact with continuous planetesimal accretion in our nominal model is a plausible accretion scenario for Earth. The dominance of planetesimal accretion and the change in the size distribution with time are naturally expected from the planet formation models. The giant impact stage was set in the time when the total mass in planetesimals remained comparable to the mass in protoplanets^42–44^. About half of giant impacts are not accretionary, but hit-and-run collisions which produce many fragments^45^. The change in size distribution from shallower to steeper is consistent with the inference that asteroids are born big and evolved through collisional cascades^46,47^. A size distribution of late accretion impactors shallower than that of our nominal model ($q \sim 2$ vs. $q = 3$) has been proposed as the origin for the lunar depletion of highly siderophile elements (HSE) relative to Earth^48^, but the long-lived magma ocean on the Moon also serves alternative explanation^41^. Our scenario, in which Earth chiefly formed from a swarm of planetesimals, is in contrast to the pebble accretion model^49^ and the idea that attributes the Late Veneer to a single big impact^50^, although impact fragments may act as small “planetesimals” to eject atmospheric N efficiently in those cases.

The timing of the volatile delivery onto the accreting terrestrial planets remains an open question^51^. We assumed homogeneous accretion of CI chondritic building blocks combined with dry objects in the nominal model and investigated the dependence on the variety of Earth’s accretion models for both the volatile-rich late accretion^9,52^ and the heterogeneous accretion model^53–55^ (Fig. 4b). The latter two models, which assumed later addition of 100% CI chondrites accretion, result in much larger amounts of volatiles, especially for N, than the current BSE inventory. This means that the volatile content fraction of the late accretion impactors appears more significant factor than the total accumulated amount for explaining Earth’s N depletion. The uncertainty in the late accretion mass (0.5–2.5 wt%^56^) is considered in Fig. 4c. Greater late accretion can erode more N and accumulate more C, but V-shaped patterns were obtained over the range of mass estimates.

## Magma ocean redox state

We explored how the redox state of the magma ocean affects the final volatile composition by considering oxidized (IW+1), intermediate (IW-2), and reduced (IW-3.5) conditions^5^ (see “Methods”). The current C–N–H depletion pattern can be obtained under the oxidized or intermediate magma ocean, while we ruled out the reduced condition (Fig. 4d). The redox state of the magma ocean, which successfully reproduces the BSE’s abundance of major volatile elements, has a relatively oxidized condition ($\log _{10} f_{{\mathrm{O}}_2} {\gtrsim } \mathrm{IW-2}$). In the reduced model, the final amount of H corresponding to 1.15 ocean mass (0.15 ocean mass in the mantle) was obtained; this is even smaller than the minimum estimate for present-day Earth’s mantle water content of > 0.56 ocean masses^4,5,17,57,58^. The behaviour of element partitioning during the main accretion stage is determined by solubilities in the magma ocean and the partitioning coefficients between silicate melt and metal liquid, both of which depend on the pressure-temperature-$f_{{\mathrm{O}}_2}$ conditions of the magma ocean. According to what is currently understood, C becomes soluble and less siderophile, N becomes insoluble, and H becomes far more soluble in silicate liquids under oxidized conditions compared with reduced conditions^5,21,22,59,60^. We note that assuming enstatite chondrites as volatile sources fits only with the oxidized model (Fig. S5).

Since the flux from the atmosphere to the core is proportional to the product of solubility and partitioning coefficient (see “Methods”), their influences on the resulting C cancelled each other out. The change in molecular masses (e.g., ${\mathrm{CO}}_2$ = 44 amu in the oxidized model to $\hbox {CH}_4$ = 16 amu in the reduced model) also influences partial pressure and, consequently, effective solubility slightly, but the influence is not significant. For H, the storage in the magma ocean is important to reproduce the current BSE abundance and so high solubility under oxidizing conditions is required. We emphasize that even when we employed smaller values for the partitioning coefficient of H as argued by a few previous high-pressure metal-silicate partitioning experiments^61,62^, the final H amount does not increase under the reduced magma ocean (see Supplementary Fig. S6, case ’m’) because the low solubility in silicate melt limits the influence of partitioning into metal on the BSE’s budget, supporting the idea of a relatively oxidized magma ocean during Earth’s accretion.

The oxidizing magma ocean has been proposed by several studies and mechanisms to oxidize the magma ocean as Earth grew have been suggested^63–65^. Crystallization of perovskite at the depth of the lower mantle induces disproportion of ferrous to ferric iron plus iron metal, the latter of which would then sink to the core, with the remnant in the mantle being oxidized (named self-oxidation^66^). The pressure effect on a fixed $\hbox {Fe}^{3+}$/$\hbox {Fe}^{\mathrm{total}}$ ratio leads to a redox gradient where the surface becomes more oxidized than the depth^65,67^. Both are natural outcomes of the formation of an Earth-sized planet. An increase in the mantle oxygen fugacity on growing Earth has been proposed by many heterogeneous accretion models to explain Earth’s bulk composition of refractory and moderately siderophile elements^54,66,68,69^. As shown in Fig. 3, the volatile distribution between the atmosphere, magma ocean, and metal soon converged towards quasi-steady state balancing of in/out fluxes of volatile elements within the first $\sim$ 20 wt% accretion. Given 20 wt% accretion being the mass required to reach the steady state for a give condition, the requirement for the redox state of the magma ocean ($\log _{10} f_{{\mathrm{O}}_2} \gtrsim \mathrm{IW-2}$) should be considered as that for the final $\sim$ 20 wt% planetesimal accretion before the giant impact. This result does not contradict the initial reduced condition followed by a more oxidized state.

## Volatile predictions for Earth’s core, bulk Venus, and Mars

Our scenario for the origin of Earth’s volatile depletion pattern is testable with the further constraints of the composition of light elements in the core. The final mass fractions in the metallic core in our nominal model, assuming an oxidized magma ocean (IW+1), were 0.9 wt%, 0.004 wt%, and 0.2 wt%for C, N, and, H, respectively. These predicted contents of light elements are within the range of each element’s content allowance and account for approximately 30% of the Earth’s core density deficit^70^. Therefore, other light elements such as oxygen, silicon, and sulphur should also contribute to the core density deficit. The relatively oxidized magma ocean required from our results may support the large contribution of oxygen^64^. With upcoming data of solubilities and partitioning coefficients, this model will provide a more accurate estimate of Earth’s core composition (see also Supplementary Information).

Our model also predicts the different depletion patterns of major volatile elements in bulk Venus and Mars. Venus might never experience the condensation of liquid water and, consequently, carbonate precipitation on the surface^28^. The lack of H and C storage leads to atmospheric loss of these elements as well as N. If atmospheric ${\mathrm{CO}}_2$ is the dominant C reservoir of bulk Venus, the total amount of C for Venus is $\sim$0.4 times the value for Earth^71^, supporting the model prediction. In contrast, the formation of $\hbox {H}_2$O and ${\mathrm{CO}}_2$ ice on early Mars^72^ allows the preferential loss of atmospheric N by impact erosion in the same manner as Earth^14^. From the analysis of SNC meteorites^73,74^, the Martian mantle is thought to be very reduced with (IW-1), and previous models have predicted a more reduced magma ocean for Mars than for Earth^75,76^. The reduced mantle would lead to a smaller storage of H in the magma ocean and a subsequent deficit of bulk H.

## Methods

We calculated the abundances of C, N, and H in Earth growing from a Mars-sized embryo via accretion of planetesimals and protoplanets with a box model considering the partitioning between reservoirs and atmospheric erosion caused by impacts. We defined two stages: main accretion and late accretion stages (e.g., the growth from 10% to 99.5% and the final 0.5% of current Earth mass, respectively) in our nominal model. The former is also called the magma ocean stage. For each finite mass step of planetesimal accretion, we solved the deterministic differential equations formally given by,

1 $$\begin{aligned} \frac{\mathrm{d} M_{\mathrm{i}}^{\mathrm{BSE}}}{\mathrm{d} M_{\mathrm{p}}} = x_{\mathrm{i}} - \sum _{\mathrm{sinks}} F^{\mathrm{i}}_{\mathrm{k}}, \end{aligned}$$

where $M_{\mathrm{i}}^{\mathrm{BSE}}$, $M_{\mathrm{p}}$, $x_{\mathrm{i}}$, and $F^{\mathrm{i}}_{\mathrm{k}}$ are the total mass of element i in BSE, planetary mass, the abundance of volatiles in impactors, and outflux per unit mass accretion by the process k, respectively. As the volatile loss processes (sinks), we considered the atmospheric escape $F_{\mathrm{esc}}$ through the full accretion and the core segregation $F_{\mathrm{core}}$ only for the magma ocean stage. For each accretion step, the element partitioning between surface and interior reservoirs is calculated by the mass balance modelling,

2 $$\begin{aligned} M_{\mathrm{i}}^{\mathrm{BSE}} = \sum _{\mathrm{j}}M_{\mathrm{i}}^{\mathrm{j}}, \end{aligned}$$

where the atmosphere, silicate melt, and suspended metal in the magma ocean in the main accretion stage, the atmosphere, ocean, and sedimentary carbonate rocks in the late accretion stage are considered as reservoirs j (see below for details). Each giant impact is treated separately from the statistically averaged planetesimal accretion. We note that we confirmed numerical convergence by changing the step size of the cumulative accreted mass in our simulations.

### Accretion model

In our model, Earth grows by accreting planetesimals and protoplanets during the main accretion phase, followed by late accretion composed only of planetesimals. For planet growth, we considered a change in the bulk density caused by pressure by using Eq. (3), which expresses the mass-radius relationship for planets with an Earth-like composition. Seager et al.^77^ showed a power-law relation between the masses and radii of solid planets by modelling their interior structures. They provided fitted formulas for rocky planets with 67.5 wt% $\hbox {MgSiO}_3$ + 32.5 wt% Fe as,

3 $$\begin{aligned} {\log {\tilde{R}} = k_1+\frac{1}{3} \log {\tilde{M}} + k_2 {\tilde{M}}^{k_3},} \end{aligned}$$

where ${\tilde{R}} = R/R_{\mathrm{s}}$ and ${\tilde{M}} = M/M_{\mathrm{s}}$ are the normalized radius and mass of terrestrial planets, $k_{\mathrm{i}}$ is the fitting constants ($k_1$ = 0.20945, $k_2 =$ 0.0804, $k_3 =$ 0.394), and *r* and $M_{\mathrm{s}}$ are the conversion factors obtained as $R_{\mathrm{s}} = 3.19\,R_{\mathrm{Earth}}$ and $M_{\mathrm{s}} = 6.41\,M_{\mathrm{Earth}}$; we used modified $R_{\mathrm{s}} = 3.29\,R_{\mathrm{Earth}}$ in our study to match Earth’s mass and radius without changing the power-law index. The growth by planetesimal accretion was investigated by a statistical method^14^ where the contribution of each impact was averaged over their size and velocity distributions. The size distribution is given by a single power-law $\mathrm {d}N/\mathrm {d}D\propto D^{-q}$, where *N*(*D*) is the number of objects of diameter smaller than *D* and the index *q* is a parameter. We assumed a shallow size distribution with $q = 2$ for the main accretion phase and a steep size distribution with $q = 3$, which corresponds to that of the present-day main belt asteroids^46^, for the late accretion phase in the nominal model, and investigated the dependence of the results by changing the power-law index (see main text). The minimum and maximum sizes are assumed to be $10^{-1.5}$ km and $10^3$ km, respectively. We assumed a Rayleigh distribution for the random velocity of impactors as modelled by Sakuraba et al.^14^, which corresponds to the Gaussian eccentricity distribution for impactors from the terrestrial planet feeding zone excited by a protoplanet^78^. Each giant impact is included in a discrete way separately from continuous planetesimal accretion.

Volatiles have been delivered by the accreted bodies that have formed Earth. The building blocks of Earth are not fully understood and so we treat the abundance of volatiles in an impactor as an unknown parameter. We considered CI chondrites containing volatile elements which accreted with dry planetesimals^11^. CI chondrites contain 2–5 wt% C^79–81^, 500–2000 ppm N^79–81^, and 0.47–1.01 wt% ppm H^79,80,82^, and their atomic ratio of C/N is 17.0 ± 3.0^15^. Enstatite chondrites contain 0.2–0.7 wt% C^80,81^, 100–500 ppm N^81^, and 90–600 ppm H^83,84^, and their C/N is 13.7 ± 12.1^15^. In our model, we assumed the reference abundances as listed in Table 1. The fraction of CI chondrites was used as a parameter and set to be 12 wt% in the nominal model (Supplementary Fig. S2). The results for cases where enstatite chondritic impactors are considered as volatile sources are also shown in the Supplementary Information (Supplementary Fig. S3 and Fig. S4).

Giant impactors would have experienced core-mantle differentiation and volatile loss by atmospheric erosion. We calculated the abundances of C, N, and H in protoplanets by running our model for the growth by planetesimal accretion in advance from 0.05 to 0.1 Earth masses and then adapted the result to the compositions of giant impactors.

We assumed that 32.5 wt% of the impactor mass is added to Earth as metallic iron regardless of the impactor type to reproduce the mass fraction of Earth’s core. The metal mass fraction is not necessarily equal to that of impactors because the former would be controlled by redox reactions (namely, the oxygen fugacity of the magma ocean), which are not explicitly modelled in our study.

### Atmospheric erosion and loss of impactors

Atmospheric erosion and loss of the impactors themselves through planetesimal accretion are computed as compiled by Sakuraba et al.^14^. The net impact-induced escaping flux per unit impactor mass is given by

4 $$\begin{aligned} F_{\mathrm{esc}}=\zeta x_{\mathrm{i}}+\eta , \end{aligned}$$

where $x_{\mathrm{i}}$ is the abundance of volatiles in the impactor, $\eta$ is the erosion efficiency of the atmosphere, and $\zeta$ is the escaping efficiency of the impactor vapor, and calculated for each element i. The erosion and escaping efficiencies for a single impact can be estimated from input impactor’s size and impact velocity. We calculated the efficiencies statistically-averaged over both impactor’s size and velocity distributions assumed in the above accretion model. We adopted the scaling laws obtained by numerical simulations for the atmospheric escape and for loss of an impactor’s vapour. Realistic numerical simulations of atmospheric erosion were given in 3D geometry by Shuvalov^85^, and in 2D cylindrical geometry by Svetsov^86^. In Sakuraba et al.^14^, the Svetsov model^86,87^ and Shuvalov model^85^ are adopted for the atmospheric erosion efficiency $\eta$ (Equation 5 in Sakuraba et al.^14^) and the impactor’s escaping efficiency $\zeta$ (Equation 8 in Sakuraba et al.^14^), respectively. The effect of oblique impacts that enhance the impact erosion is considered by including the angle-averaged factor estimated by Shuvalov model^85^. The volatiles in the fraction of impactors that avoided the loss are released to the atmosphere and then partitioned into other reservoirs. Since the atmospheric loss mass is proportional to the abundance of each species in the atmosphere, element partitioning between the atmosphere and the other solid or liquid reservoirs is important for atmospheric erosion^14^. We used the surface temperatures, 1,500 K for the main accretion stage and 288 K for the late accretion stage, given below to compute the atmospheric density and scale height, but we confirmed that the results are insensitive to the atmospheric temperature (Supplementary Fig. S1a).

For each giant impact, we calculated the atmospheric loss from the mixture of the proto-atmosphere and the impactor’s atmosphere caused by the global ground motion by using the model of Schlichting et al.^42^. We assumed Mars-sized (0.1 Earth masses) impactors whose impact velocity is 1.1 times the mutual escape velocity as commonly considered for the Moon-forming impact (see, for example, Hosono et al.^88^). We note that the estimates for the giant impact velocity has uncertainty from 1.0 to 1.2 times the escape velocity^89^. We also note that recent 3D smoothed particle hydrodynamics simulations^90,91^ suggest a lower erosion efficiency than that of Schlichting model by a factor of 3. However, we found that the dependence on the atmospheric erosion efficiency by a giant impact was negligible even if the atmospheric erosion was more or less efficient by a factor of three (Supplementary Fig. S1b) owing to the range in uncertainty arising from the velocity of a giant impact and/or the model dependency.

### Core segregation

We considered the core segregation under the solidified mantle in the magma ocean stage by excluding the segregated metal from the equilibrium partitioning calculation. Core-forming liquid metal droplets carry volatile elements from the magma ocean to the metal pond, and consequently, to the core. We assumed the suspended metal fraction in the magma ocean to be constant; then, the net segregation flux per unit impactor mass is given by

5 $$\begin{aligned} F_{\mathrm{core}}= C_{\mathrm{i}}^{\mathrm{met}} \left( x_{\mathrm{met}} - X_{\mathrm{MO}}X\mathrm{_{met}^{MO}} \right) , \end{aligned}$$

where $C_{\mathrm{i}}^{\mathrm{met}}$, $x_{\mathrm{met}}$, $X_{\mathrm{MO}}$, and $X\mathrm{_{met}^{MO}}$ are the volatile concentration in the metal, the metal fraction of the newly accreted mass (assumed to be 32.5 wt%), the melting fraction of the planet, and the metal mass fraction in the magma ocean, respectively. Our model is based upon previous studies^5,15^ that assumed that core-mantle separation is a single stage event as a simplification, but we improved the model to track time evolution through accretion, where core formation is a contentious process.

During growth by planetesimal accretion, the mass fraction of the molten magma ocean $X_{\mathrm{MO}}$ was fixed to 30 wt% of the planetary mass. This was derived from the estimated depth of the magma ocean (30%-40% of the mantle) constrained from the abundance of refractory siderophile elements (Ni and Co) in the present-day mantle^66^. A deeper magma ocean is also suggested^68^, but we confirmed that the results do not change significantly even if we consider a deeper magma ocean of up to 60 wt% (see Supplementary Fig. S1c).

As the Earth grows, the metal droplets descend through the deep magma ocean, continuously equilibrating with the silicate liquid^92^. Metal droplets that have reached the base of the magma ocean forms metal ponds and subsequently descend as large diapirs to the growing core without further equilibration with the surrounding silicate^66^. The metal in the magma ocean is assumed to be in equilibrium with the entire magma ocean and the mass fraction of the suspended metal $X\mathrm{_{met}^{MO}}$ is fixed to $10^{-6}$. This reference value is estimated from the typical timescales of metal droplets settling ($\tau _{\mathrm{rain-out}} \sim 10^1$ years^93^) and accretion of the Earth ($\tau _{\mathrm{accretion}} \sim 10^7$ years^94^) by,

6 $$\begin{aligned} X\mathrm{_{met}^{MO}} \sim (1-\zeta )x{{_{\mathrm{met}}}} M_\oplus \cdot \frac{\tau _{\mathrm{rain-out}} }{\tau _{\mathrm{accretion}} } \sim 10^{-6}. \end{aligned}$$

The settling timescale of the metal droplets was estimated from the magma ocean depth $\sim 10^2$–10$^3$ km divided by the settling velocity $\sim$0.5 cm/s^92^. In fact it evolves with time, but we confirmed that the results do not depend on the suspended metal fraction unless it exceeds 10$^{-2}$ because metal droplets carry almost the same mass of volatiles in total anyway (Supplementary Fig. S1d). The sum of remaining fraction of metal is assumed to have segregated from the magma ocean into the metal pond and, subsequently, into the core and are treated as one isolated reservoir.

In contrast to planetesimal accretion, giant impacts lead to a deeper magma ocean and incomplete mixing between the magma ocean and the core of the impactor. Since the Mars-sized moon-forming giant impact is thought to have delivered enough energy to completely melt the whole mantle^95^, we assumed a fully molten mantle after the giant impacts (see, for example, Canup^25^). Moreover, we confirmed that assuming partial melting for a giant impact does not change our results significantly (Supplementary Fig. S1c). For metal–silicate mixing in re-equilibration between the impactor’s core and proto-magma ocean, we considered turbulent entrainment by applying the formula of the metal fraction in the metal-silicate mixture to the completely molten mantle (Equation 6 in Degen et al.^96^).

The solidified mantle is assumed to be volatile free and is not considered as a reservoir because of its low capacity compared with the magma ocean (see, for example, Elkins-Tanton^10^). In terms of H and C, it is suggested that considerable amounts could be retained in the residual solid mantle as melt inclusions upon magma ocean solidification^97^, but this would not affect the final BSE inventory estimates. While we did not consider the volatile trapping by the solid silicate reservoir during the crystallization of the magma ocean, we did consider efficient trapping of outgassed H and C into the oceans and the crustal carbonate reservoirs as well as the mantle, respectively, which are included as BSE abundances. Whether H and C are trapped in the mantle or in the surface reservoirs does not affect the evolution of the BSE volatile contents. In the case of N, since the N partitioning coefficient between mantle minerals and silicate melt is smaller than unity by orders of magnitude even under high temperature^98^, almost all N would have been enriched in the melt during the crystallization and subsequently outgassed to form the early atmosphere. Hier-Majumder and Hirschmann^97^ showed that owing to extremely low solubility of $\hbox {N}_2$, N retention into the residual mantle is inefficient. For these reasons, the incorporation of volatiles into the solidified mantle does not change our conclusions.

### Equilibrium partitioning in magma ocean

We calculated the partitioning of elements between the magma ocean, core-forming alloy, and overlying atmosphere (Fig. 1a), assuming equilibrium partitioning. For the element partitioning between these three reservoirs, the mass balance equation (Eq. 2) can be written as,

7 $$\begin{aligned} M_{\mathrm{i}}^{\mathrm{BSE}} =M_{\mathrm{i}}^{\mathrm{atm}} + M_{\mathrm{i}}^{\mathrm{sil}} + M_{\mathrm{i}}^{\mathrm{met}} =\frac{4\pi R^2m_{\mathrm{i}}^{\mathrm{atm}} }{g{\bar{m}}r_{\mathrm{i}}}P_{\mathrm{i}}+C_{\mathrm{i}}^{\mathrm{sil}} M_{\mathrm{sil}} +C_{\mathrm{i}}^{\mathrm{met}} M_{\mathrm{met}} , \end{aligned}$$

where $P_i$ is the partial pressures, *g* is the gravitational acceleration given by $g = \frac{GM}{R^2}$, where *G* is the gravitational constant, ${\bar{m}}$ is the mean molecular mass of the atmosphere, $m_{\mathrm{i}}^{\mathrm{atm}}$ is the molecular mass of the volatile species, $r_{\mathrm{i}} = m_{\mathrm{i}}^{\mathrm{atm}} /m_{\mathrm{i}}$ is the mass ratio of the volatile species to the element of interest (e.g., $r_{\mathrm{C}} = {m_{{\mathrm{CO}}_2}}/{m_{\mathrm{C}}} = 44/12$ for C, $r_{\mathrm{N}} = {m_{\mathrm{N_2}}}/2{m_{\mathrm{N}}} = 1$ for N, $r_{\mathrm{H}} = m_{{\mathrm{H}}_2}{\mathrm{O}}/2{m_{\mathrm{H}}} = 18/2$ for H in the oxidized condition), $C_{\mathrm{i}}^{\mathrm{j}} = M_{\mathrm{i}}^{\mathrm{j}}/M_{\mathrm{j}}$ is the mass concentration in the silicate and metal, and $M_{\mathrm{sil}}$ and $M_{\mathrm{met}}$ are the masses of the magma ocean and the metal liquid in the magma ocean. The partition coefficient which characterizes elemental partitioning between silicate and metal can be written as,

8 $$\begin{aligned} D_{\mathrm{i}}^{\mathrm{met/sil}} = \frac{C_{\mathrm{i}}^{\mathrm{met}} }{C_{\mathrm{i}}^{\mathrm{sil}} }. \end{aligned}$$

Equilibrium between the atmosphere and the underlying silicate melt is given by a solubility law, that in many cases can be approximated by a Henrian constant,

9 $$\begin{aligned} S_i\,(x = 1, 2) = {\frac{C_{\mathrm{i}}^{\mathrm{sil}} }{P_i^{\frac{1}{x}}}}, \end{aligned}$$

where *x* is the ratio of the number of atoms between gas and solute phases for the element of interest (e.g., $x = 1$ for C and N, $x = 2$ for H, see below).

We defined three models for the redox state of the magma ocean: the oxidized ($\log _{10} f_{{\mathrm{O}}_2} \sim \mathrm{IW+1}$), intermediate (IW-2), and reduced (IW-3.5) conditions^5^, where $f_{{\mathrm{O}}_2}$ is the oxygen fugacity, $\mathrm{IW}$ is defined hereafter as $\log _{10} f_{{\mathrm{O}}_2}^{\mathrm{IW}}$, and $f_{{\mathrm{O}}_2}^{\mathrm{IW}}$ is $f_{{\mathrm{O}}_2}$ at the iron-wüstite buffer. We call the redox state of $\log _{10} f_{{\mathrm{O}}_2} \sim \log _{10} f_{{\mathrm{O}}_2}^{\mathrm{IW}} -2$ an ’intermediate’ condition because it corresponds to that estimated for a single stage core formation scenario^99^. As proposed by several studies^64,65^, we assumed an oxidized magma ocean in the nominal model, and investigated the dependence of the results by changing the redox state. The conditions for the metal-silicate equilibrium at the bottom of Earth’s magma ocean were estimated to be at $\sim$40 GPa from Ni and Co partitioning, and at $\sim$3750 K from V and Cr partitioning^54^. We assumed the partitioning coefficients thought to be applicable to these high *P*-*T* conditions mentioned above for each redox state model (Refs.^100–103^) as tabulated by Hirschmann^5^ (see Table 1). Since the dependence of the partitioning coefficients on *P*-*T*-$f_{{\mathrm{O}}_2}$ conditions has not been fully understood, we additionally investigated the sensitivity of our model by varying partitioning coefficients for a wide range suggested from the literature: $D_{\mathrm{C}}^{\mathrm{met/sil}}$ = 0.5–5670^18,22,59,62,100,101,104,105^, $D_{\mathrm{N}}^{\mathrm{met/sil}}$ = 0.003–150^16,22,23,103,106–108^ , and $D_{\mathrm{H}}^{\mathrm{met/sil}}$ = 0.2–100^61,62,102,109–111^ (Supplementary Text, Supplementary Table S1 and Supplementary Fig. S6).

We considered different atmospheric components and used the constant solubilities and partitioning coefficients for each redox state model: ${\mathrm{CO}}_2$, $\hbox {N}_2$, and $\hbox {H}_2$O for the nominal oxidized model; CO, $\hbox {N}_2$, and $\hbox {H}_2$ for the intermediate model; and $\hbox {CH}_4$, $\hbox {NH}_3$, and $\hbox {H}_2$ for the reduced model. All these species of molecules in the atmosphere are assumed by following Hirschmann^5^. As summarised in Table 1, we fixed solubilities for C and N and used the Moore model^19^ for H by following Hirschman^5^. Considering the range of solubilities reported by experimental studies, we also tested the model sensitivity for $S_{\mathrm{C}}$ = (0.002–8) $\left( \frac{P_{{\mathrm{CO}_{2}}}}{\mathrm{MPa}}\right)$ ppm^20,60,101,112,113^, $S_{\mathrm{N}}$ = (0.2–46) $\left( \frac{P_{{\mathrm{N}_{2}}}}{\mathrm{MPa}}\right)$ ppm^21,114,115^. For $S_{\mathrm{H}}$, we tested for more and less soluble cases than the Moore model by a factor of two (Supplementary Text, Supplementary Table S1, and Fig. S6). By substituting Eq. (8) and (9) to Eq. (5), we can get the relationship of $F_{\mathrm{core}}\propto C_{\mathrm{i}}^{\mathrm{met}} \propto S_{\mathrm{i}}D_{\mathrm{i}}^{\mathrm{met/sil}}$ which means that the volatile flux of core segregation is proportional to the product of solubility and the partitioning coefficient of each element i^5^.

### Late accretion stage surface environment

As we defined the late accretion as the last 0.5 wt% accretion, we examined whether the surface could melt again after the magma ocean solidification by roughly estimating the energy balance between the gravitational accretion and the planetary radiation from the oceanic surface during late accretion. The input accretional energy is estimated by $E_{\mathrm{in}} = (GM_{\mathrm{Earth}}M_{\mathrm{LA}}/R_{\mathrm{Earth}})\tau _{\mathrm{LA}}^{-1}$, where $M_{\mathrm{LA}}$ is 0.5 wt% of the Earth mass and $\tau _{\mathrm{LA}}\sim 10^8$ years^48^ is the timescale of the late accretion. The maximum outgoing planetary radiation flux is estimated by assuming the radiation limit from the saturated atmosphere ($\sim$300 W/$\hbox {m}^2$^116–118^). As a result, the latter exceeded the former by $\sim$2 orders of magnitude and confirmed that the planetary surface would not melt again during the late accretion.

We assumed the formation of the oceans and onset of the carbonate-silicate cycle (Fig. 1b) as modelled by Sakuraba et al.^14^. For the surface conditions, it is suggested that the oceans have formed within the several Myrs after the Moon-forming giant impact by calculating the timescale of the magma ocean solidification^26–28^. Furthermore, voluminous oceans underlain by a dry mantle, like that right after the magma ocean solidification, create an ideal situation to drive plate tectonics^33,119^. The carbonate-silicate cycle is believed to have stabilized the Earth’s climate over a long geological timescale against the increase in solar luminosity over the past 4 billion years^120^. As long as its negative feedbacks of temperature-dependent continental and seafloor weathering has been driven throughout Earth history as assumed here, the early climate was likely temperate, with temperatures on Earth is estimated to be 273–370 K^35,121^. Since we confirmed that dependence on the temperature within this range is negligible (the figure not shown), we assumed the present-day global mean temperature of *T* = 288 K as the reference value. We calculated the partitioning of elements between the atmosphere, oceans, and crust plus mantle. The vapour-liquid equilibrium sets an upper limit to the partial pressure of $\hbox {H}_2$O in the atmosphere, $P^{\mathrm{crit}}_{{\mathrm{H}_2\mathrm O}} = 1.7\times 10^{-2}\,\mathrm{bar}$^122^, calculated for the assumed surface temperature of 288 K. We impose the negative feedback of the carbonate-silicate cycle by simply setting an upper limit to the partial pressure of $P^{\mathrm{crit}}_{{\mathrm{CO}}_2} = 10$ bar, as expected for the steady state^121^. Neglecting the time lag to reach the steady state is justified by considering the short timescale of carbonate precipitation compared with the duration of late accretion (see main text). Atmospheric H and C in excess from the upper limits are partitioned into oceans and crust plus mantle reservoirs, respectively.

### Initial condition

We prepared the initial condition for the elemental abundances by assuming a chondritic bulk composition the same as planetesimal impactors and equilibrium partitioning between the atmosphere, the fully molten magma ocean, and the core. While this is a crude assumption that neglects the complexity of how planetary embryos formed, we have confirmed that the results are insensitive to the initial condition because the system soon evolves towards the quasi-steady state between the gain and loss of volatile elements.

## Supplementary information

Supplementary Information 1.

## Data Availability

The codes used to generate these results and the data that support the findings of this study are available at http://www.geo.titech.ac.jp/lab/okuzumi/sakuraba/Contents.html.
