# Supplementary material for: Numerous chondritic impactors and oxidized magma ocean set Earth’s volatile depletion
Source: Sci Rep. 2021 Oct 22;11:20894. doi: 10.1038/s41598-021-99240-w (PMC8536732; doi:10.1038/s41598-021-99240-w)
Supplement: Supplementary file 1 — Supplementary Information 1. [file 41598_2021_99240_MOESM1_ESM.pdf]

Numerous chondritic impactors and oxidized magma ocean set Earth's volatile depletion

Haruka Sakuraba[1,*], Hiroyuki Kurokawa[2], Hidenori Genda[2], and Kenji Ohta[1]

[1]Department of Earth and Planetary Sciences, Tokyo Institute of Technology, Ookayama, Meguro-ku, Tokyo, 152-8551, Japan
[2]Earth-Life Science Institute, Tokyo Institute of Technology, Ookayama, Meguro-ku, Tokyo, 152-8550, Japan
[*]sakuraba@eps.sci.titech.ac.jp

Supplementary Information

Building blocks

Since the origins of Earth's volatiles are unknown, we considered two types of chondrites as the sources: CI chondrites and Enstatite chondrites. The CI chondrites are the most primitive meteorites and their parent bodies have broadly been accepted as the origin of Earth's volatiles[1]. We treated the fraction of CI chondrites in total building blocks as a free input parameter, and 8 to 21 wt.% was chosen to bracket the nominal 12 wt.%, which is our best-fit model to reproduce the BSE volatile depletion pattern (Fig. S2), and to demonstrate the model behaviour. To determine the building blocks' composition in our model, we varied the CI chondrite fraction combined with completely dry bodies. As shown in Supplementary Fig. S2, Earth obtained a larger volume of volatiles as it accreted a larger amount of CI chondrites. We set the nominal fraction of CI chondrites in the building blocks to be 12 wt.%, which best reproduces the BSE volatile abundances, C/N, and C/H ratios estimated by Hirschmann[2]. The minimum case was 8 wt.%, which ended up within the uncertainties of the BSE C-N-H abundances. Adopting a higher estimate by Marty[3] requires the best-fit fraction to be ∼21 wt.%. However, the nominal value (12 wt.%) is consistent with classical models such as the Ringwood model[4], which contains 10 wt.% CI chondrites in the building blocks as well as Wänke[5] who argued 20 wt.%. Recent works also suggest a similar range of 8 wt.%–20 wt.%[6,7]. As the most important point of this study was reproducing the V-shaped relative depletion pattern by considering realistic processes to the extent of today's observational uncertainties, adopting higher BSE abundances by a factor of a few does not change our conclusions.

Enstatite chondrites are also candidates for Earth's building blocks and might have supplied Earth's major volatile elements[8]. We performed our model calculation for Enstatite chondrite-like impactors (see Supplementary Fig. S3 and S4) whose volatile abundances are listed in Table 1 (EC model) in the main manuscript. We set the same scenario as Fig. 2 and Fig. 3. Furthermore, we changed the redox state setting of the magma ocean since the relationship between them is still not comprehensively understood (Fig. S5). The current V-shaped C-N-H depletion pattern of BSE was successfully reproduced even with Enstatites chondritic building blocks when metal-silicate fractionation occurred under the oxidized condition($\log_{10} f_{O_2} \sim \log_{10} f_{O_2}^{IW} + 1$), but the requirement for the oxidization state was stricter than the nominal CI model (Fig. 4d). The final H abundance in the case where impactors of the EC model and intermediate oxidization state ($\log_{10} f_{O_2} \sim \log_{10} f_{O_2}^{IW} - 2$) was slightly smaller than the minimum estimate for BSE owing to the low content of H in the EC model. These results suggest that either accretion of H-rich materials (such as the CI model) or metal-silicate partitioning under oxidized conditions in the magma ocean (such as the EC model with the oxidized magma ocean) are required for reproducing the Earth's volatile composition.

As Enstatite chondrites' chemical composition is reduced and has a large fraction of iron relative to other chondrites, 100% Enstatite chondrite accretion may not allow for this oxidized condition. However, core formation is a self-oxidizing process[9,10], and the actual redox state of the magma ocean as a function of that of accreting bodies is not fully understood. Thus, given the limitations of our understanding, our treatment to assume the building blocks and the magma ocean redox state as independent parameters should be justified. These results suggest that core segregation and atmospheric erosion by impacts solve the discrepancy between BSE and chondrites to the extent of today's observational uncertainties.

Solubilities and partitioning coefficients

The element behaviour and the flux balance between the atmospheric erosion and the core segregation depend on the solubilities and partitioning coefficients that depend on P-T-f_{O_2} conditions. However, the exact conditions of the magma ocean have not been fully elucidated and the oxygen fugacity f_{O_2} in the magma ocean would have evolved over time[9]. Furthermore, each partitioning coefficient as a function of P-T-f_{O_2} has not been well-established yet. In the current understanding, C and H have

been found to be strongly siderophile[11,12], while N acts as a mildly lithophile to mildly siderophile[13], but these properties have not been fully achieved consensus. Given these limitations, in order to understand the physical behaviour of volatile element partitioning, we test cases where we varied each parameter separately, which help us to understand the element partitioning once these parameters are better constrained in the future. Supplementary Fig. S6 shows the dependence of the BSE volatile composition on the solubilities and partitioning coefficients.

C is generally thought to be a highly siderophile element[13–15]. C partition coefficient D_C increases linearly with decreasing f_{O_2} under relatively oxidized conditions ($\log_{10} f_{O_2} \gtrsim \log_{10} f_{O_2}^{IW} - 3$ to -1.5) but starts to decreases with decreasing f_{O_2} under more reduced conditions. As shown in Case (a), a higher D_C leads to a smaller amount of C in BSE because C transportation to the core is enhanced. C would be significantly depleted compared with the current BSE when a high D_C ($D_C = 5670$ in Case a) is assumed throughout the core formation. It has been proposed that C can be much less siderophile under high P-T conditions[16], but adopting an extremely low value $D_C = 0.5$ (Case b) still reproduce the current BSE's abundance. This is because the low solubility of C enables atmospheric erosion to buffer the effect to reduce partitioning into the core. C becomes less (more) soluble in silicate melt under more reduced (oxidized) conditions[17]. A lower S_C leads to less C partitioned into the core, but its influence is smaller compared with that of D_C in the range we investigated (Cases c and d).

N partitioning depends chiefly on f_{O_2} rather than P and T[13] – N becomes more soluble and D_N decreases with decreasing f_{O_2}[18–20]. However, with any values of D_N considered, the current N content in BSE was finally reproduced (Cases e and f), which is the same for the solubility as well (Cases g and h). The final N content in BSE was determined by the balance of the supply and loss during late accretion owing to the low D_N and S_N as discussed in the main text, and thus the results are insensitive to D_N and S_N.

H is weakly siderophile at low pressure (~20 GPa) and becomes much more strongly siderophile with increasing pressure[21], and D_H decreases with increasing f_{O_2}[22]. Under relatively oxidized conditions ($\log_{10} f_{O_2} \gtrsim \log_{10} f_{O_2}^{IW} - 2$), H is highly soluble in silicate melt[2], while H becomes less soluble under more reduced conditions. We note that varying H solubility by a factor of 2 does not change the results significantly (Cases k and l). The core formation under highly siderophile condition ($\log_{10} f_{O_2} \sim \log_{10} f_{O_2}^{IW} - 3.5$) would result in a deficit of water in BSE owing to both enhanced partitioning into the core with high D_H (Case i) and a small H storage in the magma ocean as discussed in the main text. In contrast to previous findings, some recent experiments reported a much less siderophile property at high P-T for H[23,24]. Adopting a lower partitioning coefficient leads to a higher abundance in BSE, but the results are within the estimate of H content in the current BSE (Case j).

Table S1. Parameter set of partitioning coefficients and solubilities for the parameter survey in Fig. S6. (O) and (R) are oxidized (nominal) and reduced models, which follow the setting of Hirschmann[17] as shown also in the Fig. 4d. In other cases, we varied each parameter separately within the range suggested by experimental and theoretical studies for the nominal model (see Methods). Experimental P-T-f_{O_2} conditions are as follows. i:3 GPa-1773 K-IW-1.0[25], ii:54 GPa-4100 K-IW-2.2[16], iii: 100 MPa-1473 K-IW+0.7[26], iv:1.2GPa-1673 K-IW-3.6[14], v: 5 GPa-1973 K-IW-0.66[19], vi:3 GPa-2073 K-IW-3.4[27], ix:135 GPa-4200 K[28], vii:0.1 MPa-1698 K-IW+4.1[18], viii:0.1 MPa-1698 K-IW-4.1[18], x:5 GPa-2125 K-IW-1.0[23]. Cases of (R) and (m) are performed to investigate the dependence of the final amount of water on D_H for the reduced model.

	$D_C^{met/sil}$	$S_C \left(\frac{P_{CO_2}}{MPa}\right)$ ppm	$D_N^{met/sil}$	$S_N \left(\frac{P_{N_2}}{MPa}\right)$ ppm	$D_H^{met/sil}$	$S_H \left(\frac{P_{H_2O}}{MPa}\right)^{\frac{1}{2}}$ ppm
O	500	1.6	20	1.0	6.5	M98 model[a]
a	5,670[i]	1.6	20	1.0	6.5	M98 model
b	0.5[ii]	1.6	20	1.0	6.5	M98 model
c	500	8[iii]	20	1.0	6.5	M98 model
d	500	0.002[iv]	20	1.0	6.5	M98 model
e	500	1.6	150[v]	1.0	6.5	M98 model
f	500	1.6	0.003[vi]	1.0	6.5	M98 model
g	500	1.6	20	46[vii]	6.5	M98 model
h	500	1.6	20	0.2[viii][18]	6.5	M98 model
i	500	1.6	20	1.0	100[ix]	M98 model
j	500	1.6	20	1.0	0.2[x]	M98 model
k	500	1.6	20	1.0	6.5	M98 model × 2
l	500	1.6	20	1.0	6.5	M98 model × 0.5
R	3,000	0.22	20	50	6.5	5.0
m	3,000	0.22	20	50	0.2[x]	5.0

[a] Moore et al. (1998)[29]

References

1. Dasgupta, R. & Grewal, D. S. Origin and Early Differentiation of Carbon and Asociated Life-Essential Volatile Elements on Earth. In *Deep Carbon*, 4–39 (Cambridge University Press, 2019).
2. Hirschmann, M., Withers, A., Ardia, P. & Foley, N. Solubility of molecular hydrogen in silicate melts and consequences for volatile evolution of terrestrial planets. *Earth Planet. Sci. Lett.* **345**, 38–48 (2012).
3. Marty, B. The origins and concentrations of water, carbon, nitrogen and noble gases on Earth. *Earth Planet. Sci. Lett.* **313**, 56–66 (2012).
4. Ringwood, A. Composition of the core and implications for origin of the Earth. *Geochem. J.* **11**, 111–135 (1977).
5. Wänke, H. Constitution of terrestrial planets. *Philos. Transactions Royal Soc. London. Ser. A, Math. Phys. Sci.* **303**, 287–302 (1981).
6. Budde, G., Burkhardt, C. & Kleine, T. Molybdenum isotopic evidence for the late accretion of outer Solar System material to Earth. *Nat. Astron.* **3**, 736–741 (2019).
7. Fitoussi, C., Bourdon, B. & Wang, X. The building blocks of Earth and Mars: A close genetic link. *Earth Planet. Sci. Lett.* **434**, 151–160 (2016).
8. Dauphas, N. The isotopic nature of the Earth's accreting material through time. *Nature* **541**, 521–524 (2017).
9. Wood, B. J., Walter, M. J. & Wade, J. Accretion of the Earth and segregation of its core. *Nature* **441**, 825 (2006).
10. Armstrong, K., Frost, D. J., McCammon, C. A., Rubie, D. C. & Ballaran, T. B. Deep magma ocean formation set the oxidation state of Earth's mantle. *Science* **365**, 903–906 (2019).
11. Dasgupta, R., Chi, H., Shimizu, N., Buono, A. S. & Walker, D. Carbon solution and partitioning between metallic and silicate melts in a shallow magma ocean: Implications for the origin and distribution of terrestrial carbon. *Geochimica et Cosmochimica Acta* **102**, 191–212 (2013).
12. Okuchi, T. Hydrogen partitioning into molten iron at high pressure: implications for Earth's core. *Science* **278**, 1781–1784 (1997).
13. Dalou, C., Hirschmann, M. M., von der Handt, A., Mosenfelder, J. & Armstrong, L. S. Nitrogen and carbon fractionation during core–mantle differentiation at shallow depth. *Earth Planet. Sci. Lett.* **458**, 141–151 (2017).
14. Armstrong, L. S., Hirschmann, M. M., Stanley, B. D., Falksen, E. G. & Jacobsen, S. D. Speciation and solubility of reduced C–O–H–N volatiles in mafic melt: Implications for volcanism, atmospheric evolution, and deep volatile cycles in the terrestrial planets. *Geochimica et Cosmochimica Acta* **171**, 283–302 (2015).
15. Li, Y., Dasgupta, R., Tsuno, K., Monteleone, B. & Shimizu, N. Carbon and sulfur budget of the silicate earth explained by accretion of differentiated planetary embryos. *Nat. Geosci.* **9**, 781–785 (2016).
16. Fischer, R. A., Cottrell, E., Hauri, E., Lee, K. K. & Le Voyer, M. The carbon content of Earth and its core. *Proc. Natl. Acad. Sci.* **117**, 8743–8749 (2020).
17. Hirschmann, M. M. Constraints on the early delivery and fractionation of Earth's major volatiles from C/H, C/N, and C/S ratios. *Am. Mineral.* **101**, 540–553 (2016).
18. Boulliung, J. *et al.* Oxygen fugacity and melt composition controls on nitrogen solubility in silicate melts. *Geochimica et Cosmochimica Acta* **284**, 120–133 (2020).
19. Li, Y.-F., Marty, B., Shcheka, S., Zimmermann, L. & Keppler, H. Nitrogen isotope fractionation during terrestrial core-mantle separation. *Geochem. Perspectives Lett.* **2** (2016).
20. Grewal, D. S., Dasgupta, R., Sun, C., Tsuno, K. & Costin, G. Delivery of carbon, nitrogen, and sulfur to the silicate earth by a giant impact. *Sci. advances* **5**, eaau3669 (2019).
21. Yuan, L. & Steinle-Neumann, G. Strong sequestration of hydrogen into the Earth's core during planetary differentiation. *Geophys. Res. Lett.* **47**, e2020GL088303 (2020).
22. Tagawa, S. *et al.* Experimental evidence for hydrogen incorporation into Earth's core. *Nat. communications* **12**, 1–8 (2021).
23. Clesi, V. *et al.* Low hydrogen contents in the cores of terrestrial planets. *Sci. advances* **4**, e1701876 (2018).
24. Malavergne, V. *et al.* Experimental constraints on the fate of h and c during planetary core-mantle differentiation. implications for the earth. *Icarus* **321**, 473–485 (2019).

25. Chi, H., Dasgupta, R., Duncan, M. S. & Shimizu, N. Partitioning of carbon between Fe-rich alloy melt and silicate melt in a magma ocean–implications for the abundance and origin of volatiles in Earth, Mars, and the Moon. *Geochimica et Cosmochimica Acta* **139**, 447–471 (2014).

26. Pan, V., Holloway, J. R. & Hervig, R. L. The pressure and temperature dependence of carbon dioxide solubility in tholeiitic basalt melts. *Geochimica et Cosmochimica Acta* **55**, 1587–1595 (1991).

27. Grewal, D. S. *et al.* The fate of nitrogen during core-mantle separation on Earth. *Geochimica et Cosmochimica Acta* **251**, 87–115 (2019).

28. Li, Y., Vočadlo, L., Sun, T. & Brodholt, J. P. The Earth's core as a reservoir of water. *Nat. Geosci.* **13**, 453–458 (2020).

29. Moore, G., Vennemann, T. & Carmichael, I. An empirical model for the solubility of H_2O in magmas to 3 kilobars. *american Mineral.* **83**, 36–42 (1998).

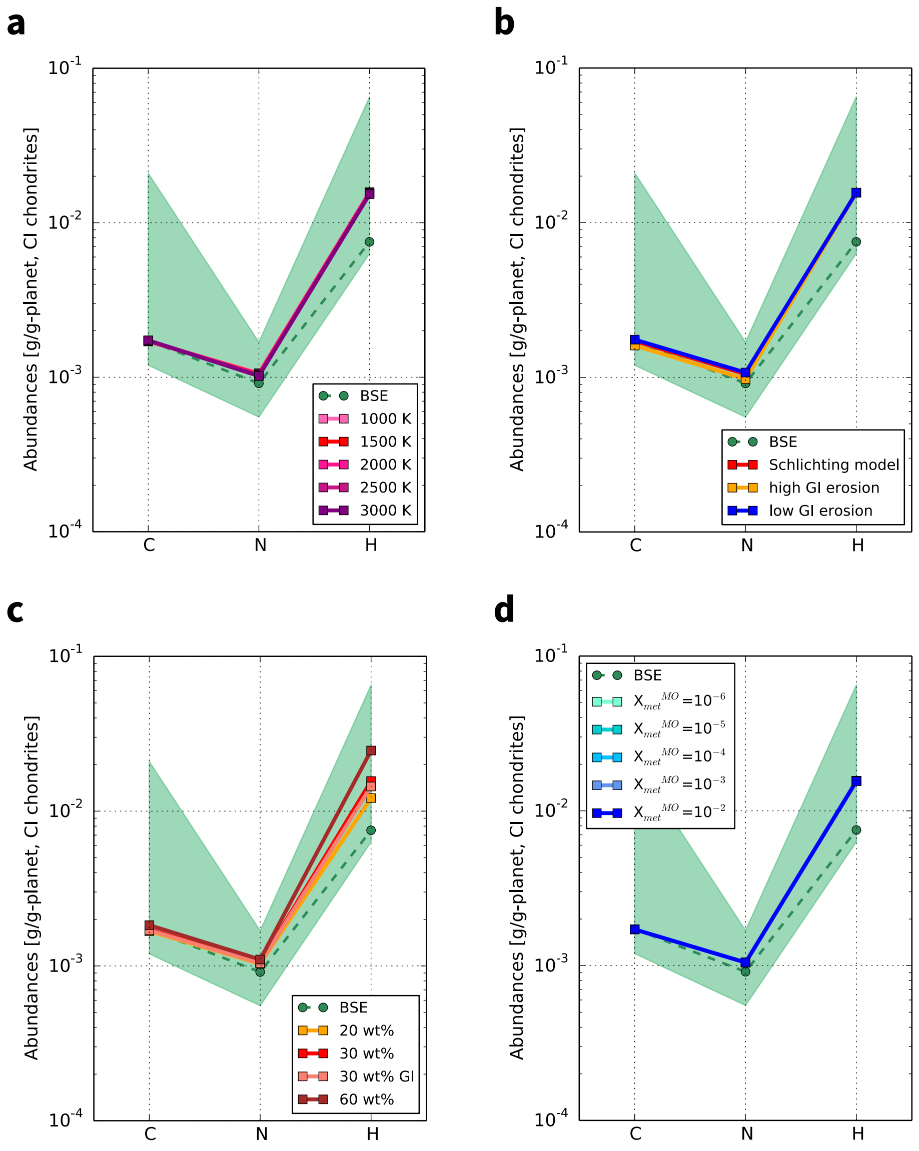

Figure S1. Dependence of final volatile composition of BSE on the parameters which have minor influences. In all cases, the other parameters are the same with the nominal model. **a.** Influence of the surface temperature on the impact-induced atmospheric erosion in our model in the main accretion. We set 1,500 K in the nominal model and varied it from 1,000 K to 3,000 K. All plots are almost overlapped. **b.** Effects of the atmospheric erosion by giant impacts. We calculated for the cases where the erosion efficiency of giant impact is three times higher and lower than Schlichting model which we used for our nominal model. **c.** Dependence on the magma ocean depth. We varied the silicate melt mass fraction on accreting Earth to 20 wt.% (orange), 30 wt.% (red), and 60 wt.% (brown). We also tested the case where a shallow magma ocean is formed after the moon-forming giant impact (pink). **d.** Dependence on the suspended metal fraction in the magma ocean. The results for different metal fractions from 10^{-6} to 10^{-2} are plotted, but all lines are overlapped.

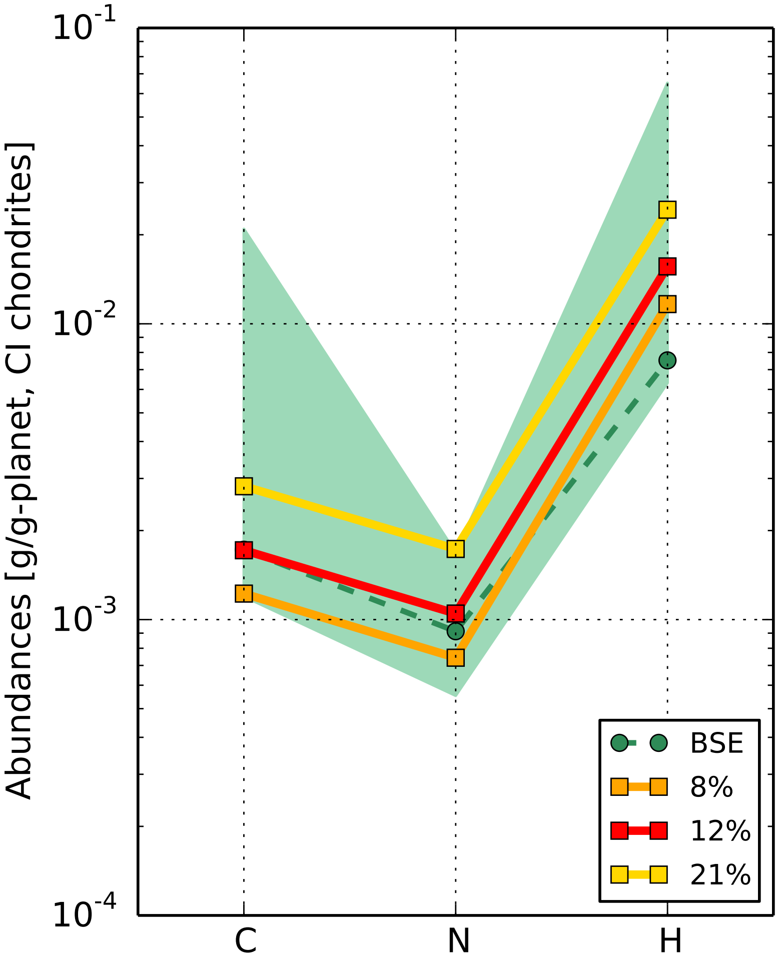

Figure S2. Dependence of final volatile composition of BSE on the CI chondrite fraction in the building blocks. The final BSE volatile abundances for cases where the CI chondrite fraction is set to be 21 wt.% (purple line), 12 wt.% (the nominal model, magenta line), and 8 wt.% (orange line) are plotted. Other parameters are the same with the nominal model. BSE's volatile abundances are the same as those in Fig. 2.

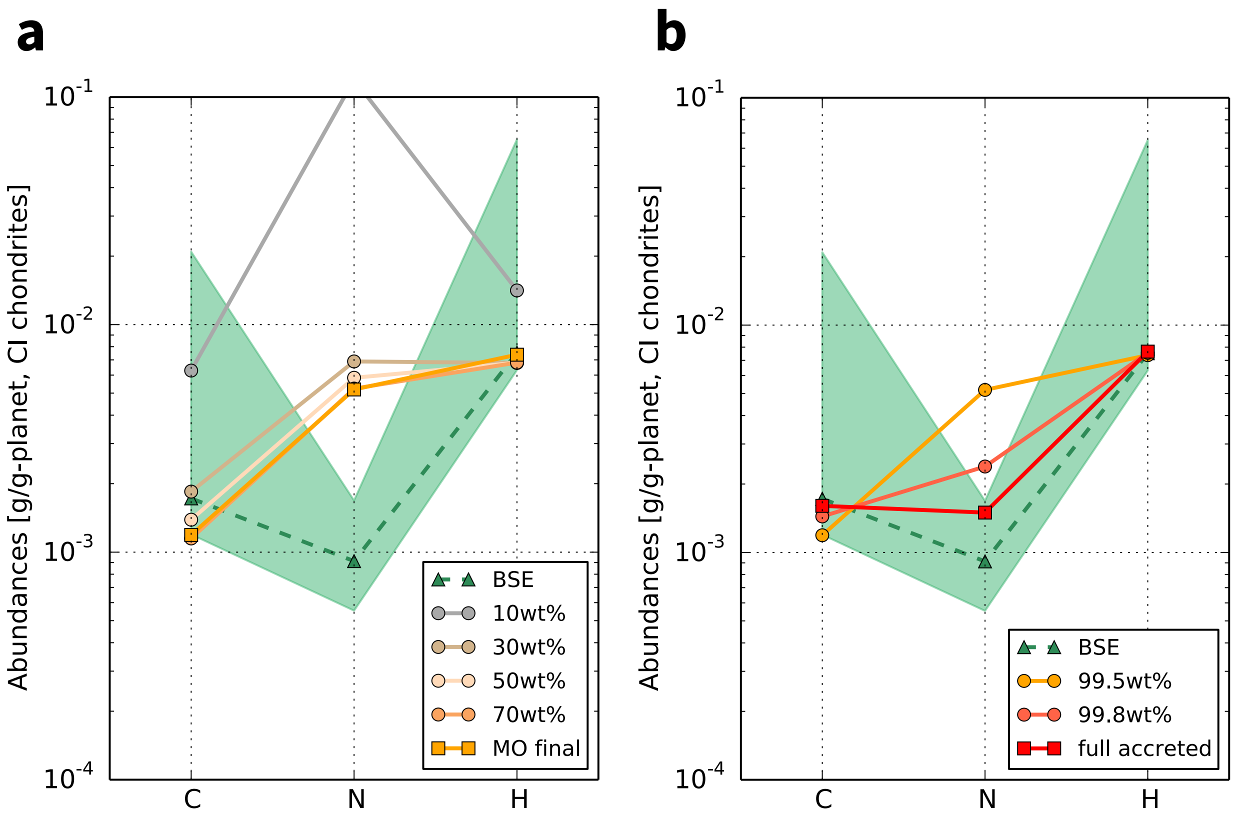

Figure S3. Results for 100% Enstatite chondrites accretion. The other parameter values are the same as those in the nominal model. This figure is equivalent to Fig. 2 for the nominal model.

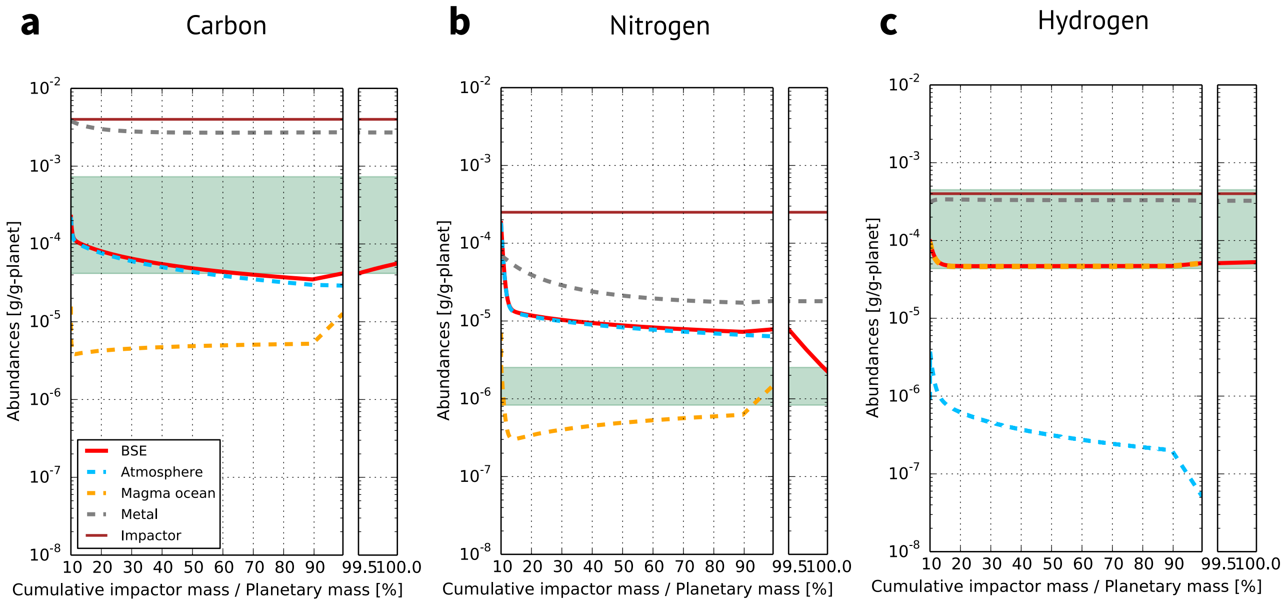

Figure S4. Results for Enstatite chondrites accretion. The other parameter values are the same as those in the nominal model. This figure is equivalent to Fig. 3 for the nominal model.

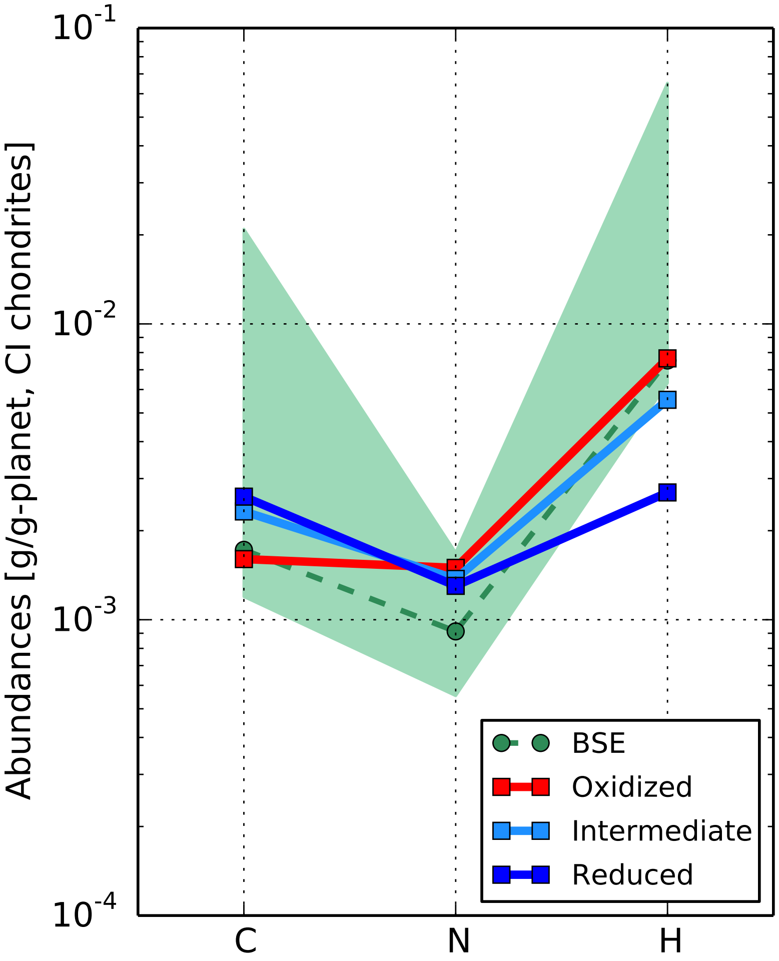

Figure S5. Dependence of final volatile composition of BSE on the redox state of the magma ocean when the building blocks are Enstatite chondrite accretion. Final BSE's volatile abundances in the cases where the building blocks are Enstatite chondrites. Redox states of the magma ocean are assumed to be oxidized (red line), intermediate (light blue line), or reduced (blue line). BSE's volatile abundances are the same with those in Fig. 2. The figure is equivalent to Fig. 4d (carbonaceous chondritic accretion). The final water abundances correspond to 2.02, 1.47, and 0.73 ocean masses in the oxidized, intermediate, and reduced cases, respectively.

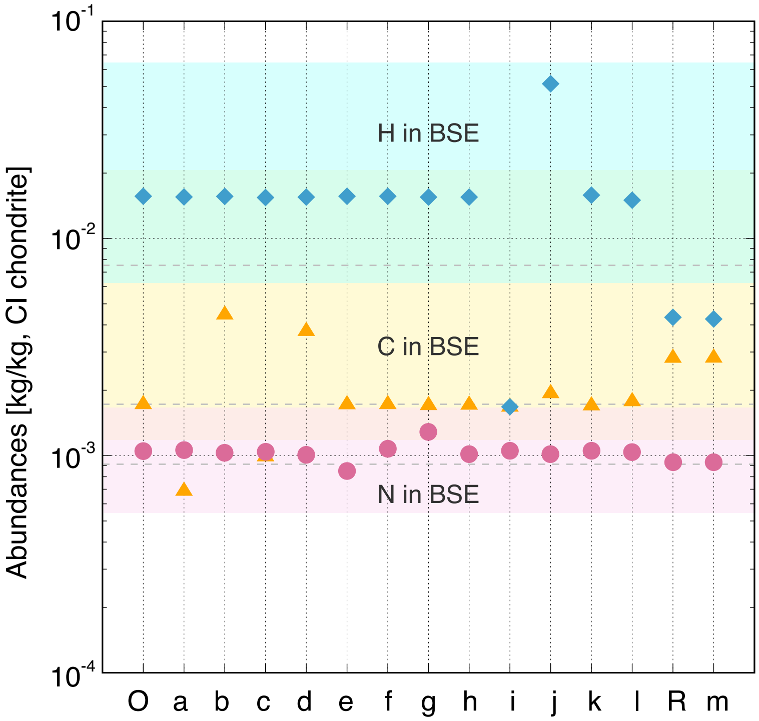

Figure S6. **Dependency of the final volatile composition in BSE on the solubilities and the partitioning coefficients.** The results of final C (orange triangles), N (magenta circles), and H (blue squares) abundances in BSE in the cases of O (oxidized nominal model), R (reduced model), a--m (Supplementary Tab. S1). The current BSE composition is shown for comparison (dashed lines and shaded areas). We varied each parameter within a wide range of values as suggested from partitioning experiments and theoretical studies as listed in Supplementary Table S1. The cases R and m show dependence on D_H in the reduced magma ocean case.
